# Risk Factors of Oral Squamous Cell Carcinoma with Special Emphasis on Areca Nut Usage and Its Association with Clinicopathological Parameters and Recurrence

**DOI:** 10.1155/2024/9725822

**Published:** 2024-08-28

**Authors:** Atif Ali Hashmi, Ghazala Mudassir, Khushbakht Rashid, Umair Arshad Malik, Shamail Zia, Fazail Zia, Muhammad Irfan

**Affiliations:** ^1^ Department of Histopathology Liaquat National Hospital and Medical College, Karachi, Pakistan; ^2^ Department of Pathology Shifa College of Dentistry Shifa Tameer-e-Millat University, Rawalpindi, Pakistan; ^3^ Department of Nephrology Sindh Institute of Urology and Transplantation, Karachi, Pakistan; ^4^ Department of Internal Medicine Aga Khan University, Karachi, Pakistan; ^5^ Department of Pathology Jinnah Sindh Medical University, Karachi, Pakistan; ^6^ Department of Biostatistics Liaquat National Hospital and Medical College, Karachi, Pakistan

## Abstract

**Introduction:**

Oral squamous cell carcinoma (OSCC) is the most prevalent type of head and neck cancer and is associated with high mortality, particularly in Southeast Asian countries. Areca nut usage, smoking, and alcohol consumption are the most common risk factors for OSCC. Areca nut chewing is highly prevalent in Pakistan and has been attributed to an increase in OSCC cases. This study aimed to determine the association between areca nut usage and various clinicopathological features of OSCC and further evaluate the association of clinicopathological parameters of OSCC with tumor recurrence.

**Materials and Methods:**

The study was conducted using the data of 228 patients with OSCC resected at Liaquat National Hospital, Karachi, Pakistan, over 5 years between 2018 and 2022. Clinicopathological data were collected from hospital archives, and associations between various risk factors and clinicopathological parameters were determined.

**Results:**

Males were more commonly affected (77.2%), and the most common age group was <50 years (54.4%). Areca nut usage was reported in 59.6% of cases, and the buccal mucosa was the most common site (62.7%). Areca nut usage was significantly associated with male gender, greater tumor size, greater depth of invasion (DOI), higher tumor stage, nodal stage, presence of perineural invasion (PNI), and recurrence. In addition, multivariate analysis revealed that OSCC recurrence was significantly associated with older age, larger tumor size and DOI, nodal metastasis, and areca nut usage.

**Conclusion:**

Areca nut-related OSCCs were associated with poor prognosis and recurrence in our study population. Furthermore, OSCC recurrence was associated with various clinicopathological parameters, such as larger tumor size, a higher DOI, and nodal metastasis.

## 1. Introduction

Oral squamous cell carcinoma (OSCC), which occurs in the oral cavity, is the most common type of head and neck carcinoma and constitutes >90% of all oral cancers [1, 2]. According to data published by Global *Cancer* Statistics, a total of 377,713 cases and 177,757 deaths of OSCC were reported globally in 2020, with the majority affecting the Asian population [3]. OSCC is associated with high mortality, with the highest mortality rate in developing countries, particularly in India and other Southeast Asian regions [4].

OSCC is more prevalent among males than females, with middle age to elderly age group being the most susceptible age group [5]. The highest incidence of OSCC is at the posterior lateral border of the tongue, accounting for 50% of all OSCC cases [6]. Tobacco smoking, betel quid (contains areca nut), and drinking alcohol are some of the most important risk factors for OSCC. Other risk factors include infection with human papilloma virus (HPV) and a diet low in fresh fruits and vegetables [7]. Areca nuts are a prominent risk factor for OSCC, and it has been estimated that over 600 million people chew areca nuts globally, and approximately 85% of this population is from Southeast Asian countries [8]. Areca nut contains four alkaloids: arecoline, arecaidine, guvacine, and guvacoline, among which arecoline exhibits carcinogenic characteristics [9].

Despite recent advancements in treatment modalities, OSCC remains a healthcare burden due to its adverse prognosis owing to its locally aggressive nature and high incidence of distant metastasis, which contributes to recurrence in approximately 30% of cases [10]. The 5-year survival rate is reported to be 92% in recurrence-free patients, which drops to 30% in patients with recurrence [11]. Evaluation of the clinicopathological features of OSCC plays a significant role in the diagnosis of the tumor, clinical outcome, and therapeutic course [12].

The chewing of areca nuts is very common in the Pakistani population and is a major risk factor for the development of OSCC. Although many studies have been conducted to evaluate the clinicopathological characteristics of OSCC, very few studies have been conducted to understand the characteristics of OSCC due to areca nut usage. This study aimed to understand the clinicopathological characteristics of areca nut-related OSCC and to determine the association of various clinicopathological parameters with recurrence.

## 2. Materials and Methods

### 2.1. Ethics, Study Design, and Setting

This was a retrospective, cross-sectional study. The study was conducted at the histopathology department of Liaquat National Hospital, Karachi, Pakistan, over 5 years from 2018 to 2022. Informed consent was obtained from all participants. All procedures were performed in accordance with the Declaration of Helsinki. This study was approved by the ethical review committee of Liaquat National Hospital.

### 2.2. Inclusion and Exclusion Criteria

All biopsy-proven cases of OSCC were included in this study. All patients included after clinical examination and workup including computed tomography (CT) scan underwent surgical treatment at the institute. Cases with missing clinicopathological or surgical data were excluded from the study. Cases with oral tumors of other types and salivary gland tumors were also excluded from the study. Patients who received neoadjuvant chemotherapy or radiotherapy were also excluded.

### 2.3. Data Collection

A total of 228 cases of OSCC that fulfilled the inclusion criteria were enrolled in the study. Clinicopathological data were retrieved from the hospital archives. The data included demographic data (gender and age), pathologic data, which included anatomical site (categorized into buccal mucosa, wet mucosa of lips, tongue, and soft palate), histological variant (keratinizing and nonkeratinizing), grade, depth of invasion (DOI) (grouped into <1 cm and >1 cm), perineural invasion (PNI), lymphovascular invasion (LVI), pTNM staging in accordance with the 8th edition of AJCC, and extra-nodal extension. Recurrence and disease-free survival were also monitored in these patients. The following risk factors were assessed: smoking, alcohol consumption, and areca nut chewing.

### 2.4. Histological Examination

The samples collected during surgery were sent to the laboratory after gross examination to determine tumor size and anatomical position. Representative sections were taken from the tumor, and hematoxylin- and eosin-stained slides were prepared. These slides were examined by a senior Histopathologist at the institute. Not all cases were reviewed by a second pathologist because all were already biopsy-proven; however, small tumors and cases with atypical histologies (that necessities immunohistochemistry) were reviewed by a second oral pathologist. Histological features like tumor differentiation, tumor grade, tumor stage, and tumor size were studied.

### 2.5. Data Analysis

The collected data were analyzed using the Statistical Package for Social Science (SPSS, Version 26.0; IBM Inc.). The mean and standard deviation for patient age, tumor size, DOI, smoking duration, areca nut chewing duration, and disease-free survival were calculated. The frequencies and percentages of all other clinicopathological variables were calculated. Chi-square and Fisher's exact tests were applied to determine the association between clinicopathological parameters and areca nut usage. Binary logistic regression was applied to determine the association between OSCC recurrence and various clinicopathological parameters.

## 3. Results

### 3.1. Clinicopathological Characteristics of Study Population

Table 1 illustrates that OSCC in our study group was more prevalent among men (77.2%) than among women (22.8%). The mean age of the patients was 50.81 ± 11.77 years, the disease being more common in the younger age group of <50 years (54.4%). The risk factor analysis showed that the majority (59.6%) of patients were areca nut chewers, with a mean duration of consumption of 15.50 ± 10.55 months. The other risk factors were fairly uncommon in our study group, with 7% of patients being smokers, with a mean smoking duration of 16.50 ± 7.24 months and alcohol consumption being reported in 1.8% of cases. The mean disease-free survival was 27.44 ± 23.51 cm. Recurrence was reported to be present in 57.9% of cases.

The most common tumor site was the buccal mucosa (62.7%), followed by the tongue (29.8%). The mean tumor size was 3.43 ± 1.74 cm. In most cases (54.4%), the tumors were of 2.1–4.0 cm in size. The mean DOI was 1.18 ± 0.77 cm. In 56.1% of cases, the DOI was <1 cm whereas in the remaining 43.9% of cases the DOI was >1 cm. Nodal metastasis was present in 50.9% of the cases. The pTNM staging of the cases depicted that the majority, approximately 36.8% cases, were at stage T2, followed by 31.6% at stage T3, whereas a minority of cases reported were at stage T1 and T4 stages (12.3% and 19.3%, respectively). The majority, approximately 49.1% of cases, were at the N0 stage, followed by 34.6% of cases at the N2b stage. Extra-nodal extension was present in 28.1% of cases. Approximately 52.6% of cases were of the keratinizing type of OSCC. The most common tumor grade was Grade 2 (moderately differentiated) in approximately 70.2% of cases. LVI was present in only 2.2% of cases and PNI was present in 19.3% of cases, as shown in Table 2.

### 3.2. Association between Areca Nut Usage and Various Clinicopathological Features

Table 3 demonstrates the association between various clinical features/risk factors and areca nut usage. The study showed a statistically significant association between areca nut chewing and gender, and recurrence. We found that males with areca nut usage were more susceptible to OSCC than those who did not use areca nut (82.4% vs. 69.6%, respectively). Areca nut usage was significantly associated with recurrence (64.7%) compared with nonareca nut-related OSCC (47.8%).

Table 4 depicts the association between pathological features and areca nut usage. We found a statistically significant association between areca nut chewing and tumor size, DOI, tumor stage, nodal stage, extra-nodal extension, histological grade, and PNI. Areca nut-related OSCC were more likely to be of larger size 2.1–4 cm compared with nonareca nut-related OSCC (55.9% vs. 52.2%, respectively), while in some cases of areca nut-related OSCC (32.4%) the size was >4 cm and about 17.4% of nonareca nut-related OSCC were of >4 cm. OSCC due to areca nut showed greater DOI compared with nonareca nut-related OSCC (DOI <1 cm 47.1% vs. 69.6%, respectively, DOI >1 cm 52.9% vs. 30.4%, respectively). Areca nuts were associated with OSCC with higher tumor stage compared with OSCC not related to areca nut (T1-8.8% vs. 17.4%, T2-32.4% vs. 43.5%, T3-32.4% vs. 30.4%, T4-26.5% vs. 8.7%, respectively). Similarly, areca nuts were associated with a higher nodal stage compared with nonareca nut-related OSCC (N2b-38.2% vs. 29.3%, N2c-2.9% vs. 5.4%, respectively). Extranodal extension was more common in OSCC not related to areca nuts than in areca nut-related OSCC (39.1% vs. 20.6%, respectively). Conversely, areca nut usage was associated with lower grade (well differentiated tumors, 29.4% in areca nut users vs. 8.7% in nonusers). PNI was more common in areca nut-related OSCC (23.5%) than in nonareca nut-related OSCC (13%). No statistically significant association was observed between areca nut usage and tumor site, nodal metastasis, histological subtype, or LVI.

### 3.3. Recurrence of OSCC and Its Association with Various Clinicopathological Features

Table 5 illustrates OSCC recurrence and its association with clinical features and risk factors by univariate and multivariate analyses. The study showed a statistically significant (*p* value <0.05) association of recurrence with age and history of areca nut usage. Keeping 95% CI and adjusting variables, we found that age >50 years and tumor size >4 cm were associated with a higher recurrence rate in both the unadjusted and adjusted groups. History of areca nut usage increased the risk of recurrence 2.0 times in the unadjusted group and 2.586 times in the adjusted group.

Table 6 depicts the association of recurrence with pathological features. We found a statistically significant association of recurrence with tumor size, DOI, nodal metastasis, tumor stage, nodal stage, and extra-nodal extension. A DOI of >1 cm was associated with a higher recurrence rate than a DOI of <1 cm (OR of 1 vs. 0.3, respectively) in the unadjusted group. Nodal metastasis was associated with recurrence with an OR of 3.5 (2.013–6.085) in the unadjusted group and an OR of 4.45 (1.853–10.723) in the adjusted group. Higher tumor stage was positively associated with recurrence (OR: T1-0.296, T2-0.111, and T4-1). Similarly, higher nodal stage was associated with recurrence, i.e., N2b showed 4.211 times and N2c showed 10.667 times greater risk of recurrence in the unadjusted group. No statistically significant association was established between OSCC recurrence and tumor site, keratinizing type, histological grade, LVI, or PNI.

### 3.4. Comparison of Recurrence-Free Survival between Patients with and without a History of Areca Nut Use

**Figure 1:**
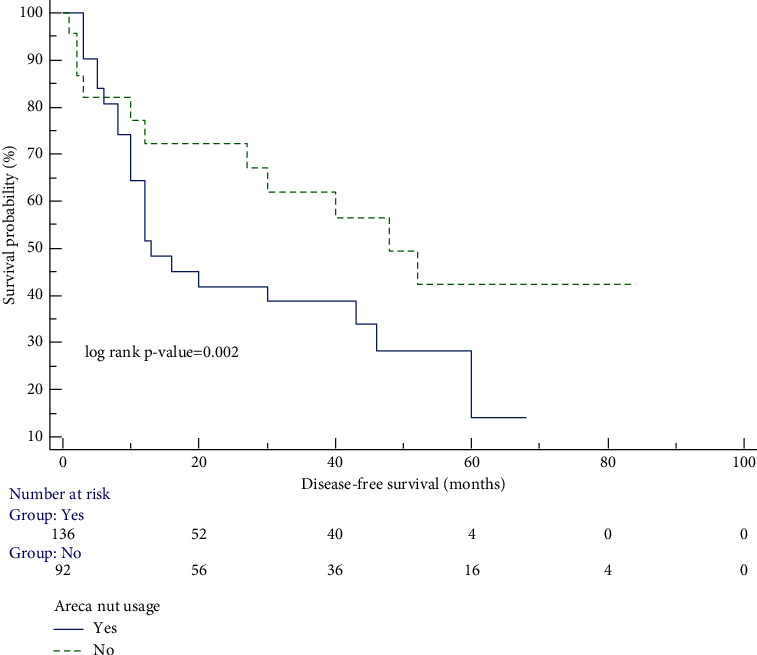
Survival analysis of oral carcinoma patients (areca nut usage vs. no areca nut use) by the Kaplan–Meier method.

Figure 1 demonstrates the survival curve using the Kaplan–Meier graph, which shows that recurrence-free survival among patients with a history of areca nut use was significantly lower than that among patients with no areca nut use.

### 3.5. Association of Duration of Areca Nut Use with Clinicopathological Parameters

Table 7 shows the mean comparison of the duration of areca nut usage with the main prognostic parameters, including recurrence, tumor stage, nodal metastasis, PNI, and DOI. No significant association was noted between the duration of areca nut usage and these parameters.

## 4. Discussion

OSCC is the most common malignant tumor of the head and neck and is a global health issue because of its aggressive nature [13]. This study determined the clinicopathological features of the disease in our study population, its recurrence, and the association of clinicopathological features with areca nut.

A study conducted in Qatar by Elaiwy et al. [14] to determine the pathological features of OSCC concluded that similar to our study, males were predominantly more affected than females. The mean age in their study was reported to be 46.93 years, whereas in our study the mean age was 50.81 ± 11.77 years which corroborates with the data reported previously that most OSCC patients are >45 years (median 62 years) [15]. The most common site in their study was tongue (50%), followed by the buccal mucosa, whereas in our study, the buccal mucosa was the most common site of OSCC (62.7%). The mean DOI in their study was 8.8 mm, whereas in our study the mean DOI was slightly greater 1.18 ± 0.77 cm. Similar to our findings, the most common histological grade was Grade 2, and most cases showed no LVI or PNI.

This study was conducted to evaluate the clinicopathological features associated with areca nut-related OSCC. We concluded that areca nut usage was significantly associated with male gender, greater tumor size (2.0– >4 cm), DOI of >1 cm (52.9%), higher tumor stage (T3 and T4-32.4% and 26.5%, respectively), higher nodal stage (N2b-38.2%), and a higher histological grade (Grade 2 and Grade 3–61.8% and 8.8%, respectively). Areca nut-related OSCC was more likely to show PNI (23.5%) than nonareca nut-related OSCC (13%). The recurrence rate of areca nut-related OSCC in our study was 64.7%, which was much higher than that of OSCC not related to areca nut (47.8%).

Clinicopathological features associated with areca nut use remain largely unexplored. A few studies have been conducted to determine the association between various clinicopathological parameters of OSCC and areca nut usage, but most failed to report a positive association.

A similar study was conducted in China to determine the correlation between betel nut chewing and clinicopathological factors of OSCC. In contrast to our study, they reported no significant correlation between betel nut chewing and gender, age, location, pathological T stage, and cervical lymph node metastasis [16].

In accordance with our study, a study conducted in Pakistan to determine the role of chewing habits in the differentiation of OSCC found that patients with chewing habits were associated with poorly differentiated (Grade 3) tumors and were of younger age [17]. In our study, no association was found between age and areca nut chewing. Another study conducted in Northern Pakistan, similar to our study, reported an association between male gender and betel nut chewing [18].

A study conducted by Li et al. on multifaceted mechanism of areca nut in oral carcinogenesis, proposed that males with areca nut chewing habits were more likely to develop OSCC (especially of buccal mucosa), and are of aggressive phenotype, with a greater risk of metastasis, a higher recurrence rate, and a poor survival rate [19]. These findings were supported by our study, which reported that areca nut usage was significantly associated with male gender and a high recurrence rate, with a recurrence rate of 64.7%. Another study by Liao et al. [20] also corroborated that OSCC in habitual areca nut chewers showed an aggressive clinical course. Similar to our finding, a study conducted in Taiwan reported that 49.3% of OSCC patients who consumed areca nut usage presented with a later stage tumor (T3-T4) [21].

Local recurrence is an important prognostic factor in OSCC [10]. Our study also evaluated the association of OSCC recurrence with clinicopathological parameters, and we concluded that OSCC recurrence showed a strong association with age of >50 years, tumor size of >4 cm, DOI of >1 cm, nodal metastasis, higher tumor and nodal stage, and history of pan.

PNI is strongly associated with recurrence and distant metastasis in OSCC [22]. This study failed to establish a statistically significant association between PNI and OSCC recurrence. Wang et al. [10] reported a significant association between tumor grade and recurrence, which our study failed to establish. A previous study reported that margin status was the only independent predictor of recurrence, whereas DOI and nodal status were important prognostic factors of survival [23]. Wang et al. [10] also concluded that T stage, nodal stage, and degree of differentiation were independent factors of recurrence. Tumor and nodal stages are important factors affecting recurrence, which is also supported by a study by Ebrahimi et al. [24].

Apart from areca-nut use, various other risk factors are also established in OSCC pathogenesis, including the emerging use of e-cigarettes/vaping. Several known carcinogens are found in e-cigarettes, and studies have shown certain molecular alterations associated with e-cigarettes, including DNA strand breaks, that are potentially linked to oral cancers [25]. In addition to nicotine in its various forms, human papilloma virus (HPV) is another strongly associated risk factor involved in OSCC. Approximately 20% of OSCCs are attributed to HPV. Conversely, HPV-associated OSCCs are associated with better prognostic features than nicotine/betal-nut-associated OSCC [13].

As established in our study and supported by other reports, it is evident that areca-nut use is one of the most important risk factors for OSCC in Southeast Asia. Therefore, it is imperative to devise measures to spread awareness in the public to restrict its use, maintain good oral hygiene, and seek early medical advice in cases with any oral ulcer or nodule.

### 4.1. Limitations

We recognized a few limitations of this study. First, this was a single institute-centered study; hence, the sample size was limited. This study lacks a comprehensive molecular study. The study is retrospective. A multicenter prospective study is imperative to understand the disease and to modulate newer treatment modalities to improve overall survival and prognosis.

Because of the retrospective study design, certain aspects of the link between OSCC occurrence and areca nut usage could not be uncovered in our study. First, in this study, chronically addicted areca-nut patients were labeled as areca nut users, and this history was obtained when these patients presented in the ENT OPD. The degree of areca nut usage was not quantified; however, the duration of use was evaluated, and it was noted that the mean duration of area nut usage in our study was 15.50 ± 10.55 months. This specifies that unlike other risk factors, such as smoking, even a small duration of areca nut usage can cause ORCC. However, how much areca nut could be safe could not be determined by our study design, as there was no control group (without OSCC) in our study. A prospective cohort study is required to further establish the risk of OSCC associated with areca-nut use.

Similarly, it is also important to understand the molecular alterations that are specifically associated with areca-nut use to devise new treatment modalities for ORCC in this part of the world.

## 5. Conclusion

We found that areca nut-related OSCC was significantly associated with various clinicopathological features, including gender, tumor size, DOI, T stage, nodal stage, PNI, and recurrence. Furthermore, we concluded that age, tumor size, DOI, nodal metastasis, tumor stage, nodal stage, and history of areca nut usage were important factors for recurrence.

## Figures and Tables

**Table 1:** Clinical parameters and risk factors of population under study.

Clinical parameters	Values
*Gender*	
Male, *n* (%)	176 (77.2)
Female, *n* (%)	52 (22.8)

Age (years); mean ± SD	50.81 ± 11.77

*Age groups*	
≤50 years, *n* (%)	124 (54.4)
>50 years, *n* (%)	104 (45.6)

Disease-free survival (months); mean ± SD	27.44 ± 23.51

Risk factors	

*Smoking*	
Smokers, *n* (%)	16 (7)
Nonsmokers, *n* (%)	212 (93)
Smoking duration (months); mean ± SD (*n* = 16)	16.50 ± 7.24

*Alcohol intake*	
Yes, *n* (%)	4 (1.8)
No, *n* (%)	212 (93)

*Areca nut usage (pan/gutka)*	
Yes, *n* (%)	136 (59.6)
No, *n* (%)	92 (40.4)
Duration of areca nut usage (months); mean ± SD (*n* = 136)	15.50 ± 10.55

*Recurrence*	
Yes, *n* (%)	132 (57.9)
No, *n* (%)	96 (42.1)

**Table 2:** Pathological parameters of population under study.

Pathological parameters	Values
Tumor size (cm); mean ± SD	3.43 ± 1.74

*Tumor size groups*	
≤2 cm, *n* (%)	44 (19.3)
2.1–4.0 cm, *n* (%)	124 (54.4)
>4 cm, *n* (%)	60 (26.3)

Depth of invasion (cm); mean ± SD	1.18 ± 0.77

*Depth of invasion groups*	
≤1 cm, *n* (%)	128 (56.1)
>1 cm, *n* (%)	100 (43.9)

*Tumor site*	
Buccal mucosa, *n* (%)	143 (62.7)
Wet mucosa of lip, *n* (%)	5 (2.2)
Tongue, *n* (%)	68 (29.8)
Soft palate, *n* (%)	12 (5.3)

*Nodal metastasis*	
Present, *n* (%)	116 (50.9)
Absent, *n* (%)	112 (49.1)

*Tumor (T) stage*	
T1, *n* (%)	28 (12.3)
T2, *n* (%)	84 (36.8)
T3, *n* (%)	72 (31.6)
T4, *n* (%)	44 (19.3)

*Nodal (N) stage*	
N0, *n* (%)	112 (49.1)
N1, *n* (%)	28 (12.3)
N2b, *n* (%)	79 (34.6)
N2c, *n* (%)	9 (3.9)

*Extra-nodal extension*	
Present, *n* (%)	64 (28.1)
Absent, *n* (%)	164 (71.9)

*Histological subtype*	
Nonkeratinizing, *n* (%)	108 (47.4)
Keratinizing, *n* (%)	120 (52.6)

*Histologic grade*	
Grade 1/well differentiated, *n* (%)	48 (21.1)
Grade 2/moderately differentiated, *n* (%)	160 (70.2)
Grade 3/poorly differentiated, *n* (%)	20 (8.8)

*Lymphovascular invasion*	
Present, *n* (%)	5 (2.2)
Absent, *n* (%)	223 (97.8)

*Perineural invasion*	
Absent, *n* (%)	184 (80.7)
Present, *n* (%)	44 (19.3)

**Table 3:** Association of clinical parameters and other risk factors with areca nut usage.

Clinical parameters/risk factors	Values		*p* value
	Areca nut usage		
	Yes	No	
*Gender* ^∗^			
Male, *n* (%)	112 (82.4)	64 (69.6)	0.024^∗∗∗^
Female, *n* (%)	24 (17.6)	28 (30.4)	

*Age groups* ^∗^			
≤50 years, *n* (%)	80 (58.8)	44 (47.8)	0.102
>50 years, *n* (%)	56 (41.2)	48 (52.2)	

*Smoking* ^∗^			
Smokers, *n* (%)	8 (5.9)	8 (8.7)	0.415
Nonsmokers, *n* (%)	128 (94.1)	84 (91.3)	

*Recurrence* ^∗^			
Yes	88 (64.7)	44 (47.8)	0.011^∗∗∗^
No	48 (35.3)	48 (52.2)	

^∗^Chi-square test was applied. ^∗∗^Fisher exact test was applied. ^∗∗∗^Significant at 0.05 level.

**Table 4:** Association of pathological parameters with areca nut usage.

Pathological parameters	Values		*p* value
	Areca nut usage		
	Yes	No	
*Tumor size groups* ^∗^			
≤2 cm, *n* (%)	16 (11.8)	28 (30.4)	<0.001^∗∗∗^
2.1–4.0 cm, *n* (%)	76 (55.9)	48 (52.2)	
>4 cm, *n* (%)	44 (32.4)	16 (17.4)	

*Depth of invasion groups* ^∗^			
≤1 cm, *n* (%)	64 (47.1)	64 (69.6)	<0.001^∗∗∗^
>1 cm, *n* (%)	72 (52.9)	28 (30.4)	

*Tumor site* ^∗∗^			
Buccal mucosa, *n* (%)	84 (61.8)	59 (64.1)	0.845
Wet mucosa of lip, *n* (%)	4 (2.9)	1 (1.1)	
Tongue, *n* (%)	40 (29.4)	28 (30.4)	
Soft palate, *n* (%)	8 (5.9)	4 (4.3)	

*Nodal metastasis* ^∗^			
Present, *n* (%)	64 (47.1)	52 (56.5)	0.161
Absent, *n* (%)	72 (52.9)	40 (43.5)	

*Tumor (T) stage* ^∗^			
T1, *n* (%)	12 (8.8)	16 (17.4)	0.003^∗∗∗^
T2, *n* (%)	44 (32.4)	40 (43.5)	
T3, *n* (%)	44 (32.4)	28 (30.4)	
T4, *n* (%)	36 (26.5)	8 (8.7)	

*Nodal stage* ^∗∗^			
N0, *n* (%)	72 (52.9)	40 (43.5)	0.002^∗∗∗^
N1, *n* (%)	8 (5.9)	20 (21.7)	
N2b, *n* (%)	52 (38.2)	27 (29.3)	
N2c, *n* (%)	4 (2.9)	5 (5.4)	

*Extra-nodal extension* ^∗∗^			
Present, *n* (%)	28 (20.6)	36 (39.1)	0.002^∗∗∗^
Absent, *n* (%)	108 (79.4)	56 (60.9)	

*Histological subtype* ^∗^			
Nonkeratinizing, *n* (%)	60 (44.1)	48 (52.2)	0.232
Keratinizing, *n* (%)	76 (55.9)	44 (47.8)	

*Histologic grade* ^∗^			
Grade 1/well differentiated, *n* (%)	40 (29.4)	8 (8.7)	<0.001^∗∗∗^
Grade 2/moderately differentiated, *n* (%)	84 (61.8)	76 (82.6)	
Grade 3/poorly differentiated, *n* (%)	12 (8.8)	8 (8.7)	

*Lymphovascular invasion* ^∗∗^			
Present, *n* (%)	4 (2.9)	1 (1.1)	0.651
Absent, *n* (%)	132 (97.1)	91 (98.9)	

*Perineural invasion* ^∗^			
Present	32 (23.5)	12 (13)	0.049^∗∗∗^
Absent	104 (76.5)	80 (87)	

^∗^Chi-square test was applied. ^∗∗^Fisher exact test was applied. ^∗∗∗^Significant at 0.05 level.

**Table 5:** Recurrence of oral squamous cell carcinoma, comparison with groups based on clinical features/risk factors: univariate and multivariate analysis.

Clinical parameters/risk factors	Unadjusted variables		Adjusted variables	
	*p* value	Values	*p* value	Values
*Gender*				
Male, odds ratio (95% CI)	0.545	0.822 (0.437–1.549)		
Female®		1		

*Age groups*				
≤50 years, odds ratio (95% CI)	0.037^∗∗∗^	0.565 (0.330–0.965)	<0.001^∗∗∗^	0.212 (0.103–0.439)
>50 years®		1		1

*Smoking*				
Smokers, odds ratio (95% CI)	0.509	0.710 (0.257–1.963)		
Nonsmokers®		1		

*History of areca nut usage*				
Yes, odds ratio (95% CI)	0.012^∗∗∗^	2.000 (1.166–3.430)	0.008^∗∗∗^	2.586 (1.285–5.203)
No®		1		1

Binary logistics regression was applied. CI: confidence interval, ®reference group. ^∗∗∗^Significant at 0.05 level.

**Table 6:** Recurrence of oral squamous cell carcinoma, comparison with different pathological groups: univariate and multivariate analysis.

Clinicopathological groups	Unadjusted variables		Adjusted variables	
	*p* value	Values	*p* value	Values
*Tumor size groups*				
≤2 cm, *n* (%)	<0.001^∗∗∗^	0.128 (0.049–0.332)	0.004^∗∗∗^	0.177 (0.055–0.566)
2.1–4.0 cm, *n* (%)	<0.001^∗∗∗^	0.144 (0.063–0.329)	<0.001^∗∗∗^	0.135 (0.053–0.344)
>4 cm, *n* (%)		1		1

*Depth of invasion groups*				
≤1 cm, odds ratio (95% CI)	<0.001^∗∗∗^	0.343 (0.196–0.599)	0.279	0.684 (0.345–1.359)
>1 cm®		1		1

*Tumor site*				
Buccal mucosa, odds ratio (95% CI)	0.726	0.800 (0.230–2.783)		
Wet mucosa of lip, odds ratio (95% CI)	0.587	2.00 (0.164–24.328)		
Tongue, odds ratio (95% CI)	0.218	0.444 (0.122–1.617)		
Soft palate®		1		

*Nodal metastasis*				
Present, odds ratio (95% CI)	<0.001^∗∗∗^	3.500 (2.013–6.085)	<0.001^∗∗∗^	4.458 (1.853–10.723)
Absent®		1		1

*Tumor (T) stage*				
T1, odds ratio (95% CI)	0.026^∗∗∗^	0.296 (0.102–0.865)		
T2, odds ratio (95% CI)	<0.001^∗∗∗^	0.111 (0.046–0.271)		
T3, odds ratio (95% CI)	0.244	0.578 (0.229–1.455)		
T4®		1		

*Nodal (N) stage*				
N1, odds ratio (95% CI)	0.178	1.778 (0.770–4.105)		
N2b, odds ratio (95% CI)	<0.001^∗∗∗^	4.211 (2.226–7.964)		
N2c, odds ratio (95% CI)	0.028^∗∗∗^	10.667 (1.290–88.181)		
N0®		1		

*Extra-nodal extension*				
Present, odds ratio (95% CI)	<0.001^∗∗∗^	0.350 (0.184–0.666)	0.984	0.990 (0.367–2.672)
Absent®		1		1

*Keratinizing*				
Nonkeratinizing, odds ratio (95% CI)	0.142	1.487 (0.875–2.528)		
Keratinizing®		1		

*Histologic grade*				
Grade-I, odds ratio (95% CI)	0.899	0.933 (0.322–2.702)		
Grade-II, odds ratio (95% CI)	0.831	0.902 (0.350–2.327)		
Grade-iii®		1		

*Lymphovascular invasion*				
Present, odds ratio (95% CI)	0.122	0.176 (0.019–1.596)		
Absent®		1		

*Perineural invasion*				
Present, odds ratio (95% CI)	0.391	1.346 (0.682–2.657)		
Absent®		1		

Binary logistics regression was applied. CI: confidence interval, ®reference group. ^∗∗∗^Significant at 0.05 level.

**Table 7:** Association of areca nut usage duration with clinicopathological parameters.

Clinicopathological parameters	Values	*p* value
	Areca nut usage duration (months)	
*Recurrence*		
Yes; mean ± SD	16.090 ± 11.438	0.379
No; mean ± SD	14.416 ± 8.717	

*Nodal metastasis*		
Present; mean ± SD	14.750 ± 6.156	0.437
Absent; mean ± SD	16.166 ± 13.314	

*Perineural invasion*		
Present; mean ± SD	17.875 ± 16.643	0.314
Absent; mean ± SD	14.769 ± 7.770	

*Tumor (T) stage*		
T1/T2; mean ± SD	15.521 ± 12.032	0.972
T3/T4; mean ± SD	15.454 ± 6.58	

*Depth of invasion*		
≤1 cm; mean ± SD	16.750 ± 14.144	0.215
>1 cm; mean ± SD	14.388 ± 5.628	

Independent *t*-test was applied, SD: standard deviation.

## Data Availability

The data that support the findings of this study are available from the corresponding author upon reasonable request.
